# Craniopagus parasiticus – a parasitic head protruding from temporal area of cranium: a case report

**DOI:** 10.1186/s13256-016-1023-3

**Published:** 2016-12-01

**Authors:** Wassihun Nega, Meku Damte, Yonas Girma, Getachew Desta, Mengistu Hailemariam

**Affiliations:** 1 Department of Obstetrics and Gynecology, Bahir Dar University, Bahir Dar, Ethiopia; 2 Department of Surgery, Bahir Dar University, Bahir Dar, Ethiopia; 3 Center of International Reproductive Health Training (CIRHT), Bahir Dar University, Bahir Dar, Ethiopia; 4 Center of International Reproductive Health Training (CIRHT) Manager, Addis Ababa, Ethiopia

**Keywords:** Craniopagus parasiticus, Parasitic twin, Rare case

## Abstract

**Background:**

Craniopagus parasiticus is rare with an incidence of approximately four to six cases in 10,000,000 births. In our case, the head of the parasitic twin protruded from the temporal area of the normal twin’s cranium. The parasitic twin had two deformed lower limbs, of which one was rudimentary, and long bones of the bilateral lower limbs and some pelvic bone. Dissection of the mass of the parasitic twin’s body revealed the intestine but no chest organs or abdominal organs. There was a rudimentary labium but no vaginal opening. In resource-limited countries, maternal age or nutritional factors may play a role in craniopagus parasiticus.

**Case presentation:**

A 38-year-old multigravida (gravida V para IV) woman of Amhara ethnicity was referred from a rural health center to our hospital due to prolonged second stage of labor at 42+1 weeks. On her arrival at our hospital, an obstetrician decided to do a caesarean section because she was unable to deliver vaginally. A live baby girl weighing 4200 g was delivered. The placenta was single and normal. Her Appearance, Pulse, Grimace, Activity, and Respiration scores were 7 and 9 at 1 and 5 minutes, respectively. She appeared to be grossly normal except for the parasitic co-twin attached to her cranium. After a week of extensive counselling and investigation, a successful separation operation was done. Postoperation, she comfortably suckled on the breast and had no neurological deficit. Two weeks after separation she was discharged in a good healthy condition with an arrangement for postnatal follow up.

**Conclusions:**

The causes of craniopagus parasiticus are still unknown due to a rarity of cases and a limited number of studies on it. There have been only nine to ten cases of craniopagus parasiticus, of which only three survived past birth and were documented in the literature. Genetic scientists and researchers continue to investigate this case because they might find explanations for the birth defect, and provide answers to improve the prognosis and the life chances of twins with craniopagus parasiticus.

## Background

Craniopagus parasiticus is extremely rare, it occurs in approximately four to six births out of 10,000,000 births [1]. In this parasitic twin type, the head of a fully formed body is connected at the temporal area with the head of a parasitic twin with an undeveloped body [2, 3]. Only ten cases of craniopagus parasiticus have been documented in the literature [4]. Most babies with craniopagus parasiticus are stillbirths but three documented cases survived birth with the help of modern medicine [5, 6]. We report the case of a baby girl delivered alive with a parasitic co-twin and a successful separation performed 1 week later.

## Case presentation

A 38-year-old multigravida (gravida V para IV) woman of Amhara ethnicity was referred from a health center to our hospital due to prolonged second stage of labor at 42+1 weeks. She felt that her pregnancy did not differ from her previous pregnancies. She had been taking injectable contraception for 2 years.

She had no family history of any congenital anomalies. She had four healthy live births at term and all are healthy. She had antenatal follow up for four visits where she was screened for human immunodeficiency virus (HIV), syphilis, hepatitis B virus (HBV), and for diabetes (only a random blood sugar test) but not sonographic screening. She received tetanus vaccination and iron supplementation. She did not take any other medication during her pregnancy. She presented to our hospital after laboring for approximately 35 hours both at home and at the health center. She was evaluated on arrival at our hospital; she had contraction, term-sized gravid uterus, and fetal heart beat was 112. On digital pelvic examination her cervix was fully dilated, the station of the head was high, and the pulsating umbilical cord was in front of the presenting part with ruptured membrane, which indicated a difficult transvaginal delivery. For this reason, the team rushed for emergency cesarean section.

**Fig. 1:**
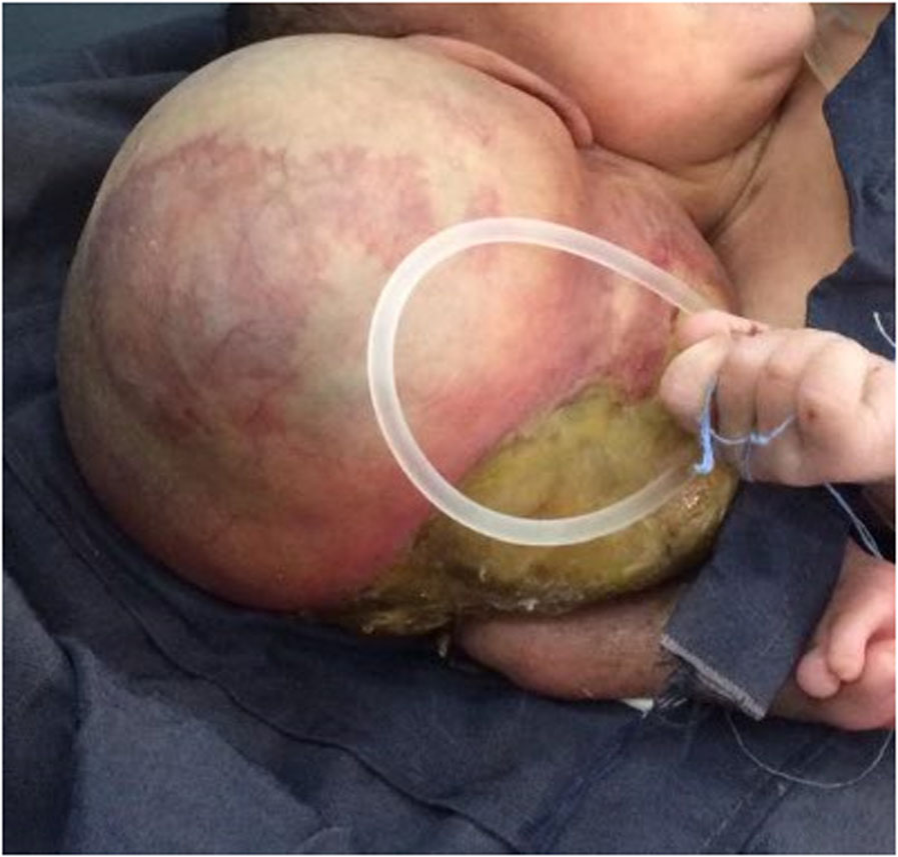
Anterior view Craniopagus parasitic twin after delivery

**Fig. 2:**
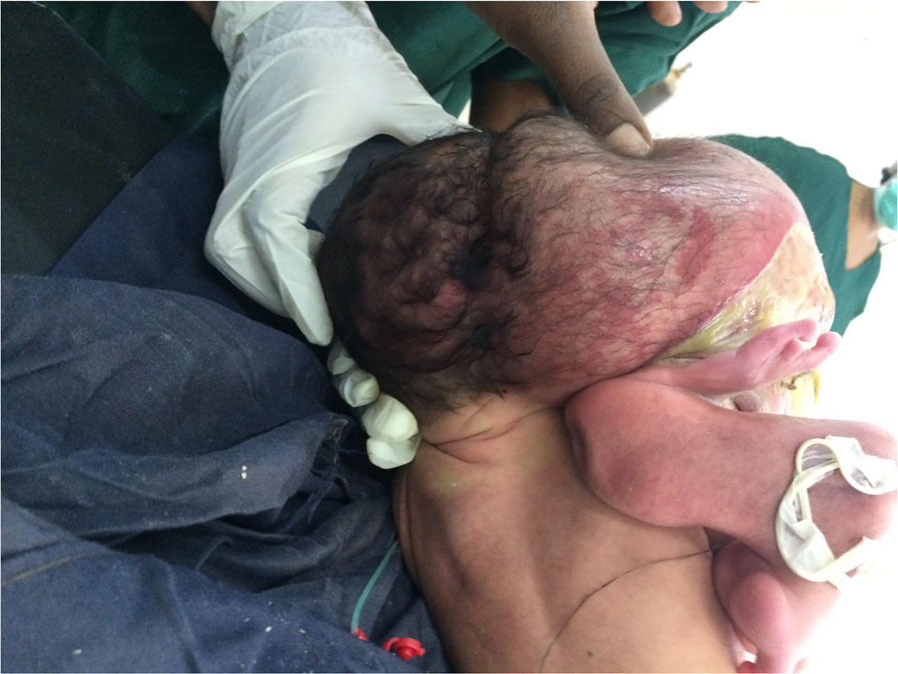
Posterior view of craniopagus parasitic twins

**Fig. 3:**
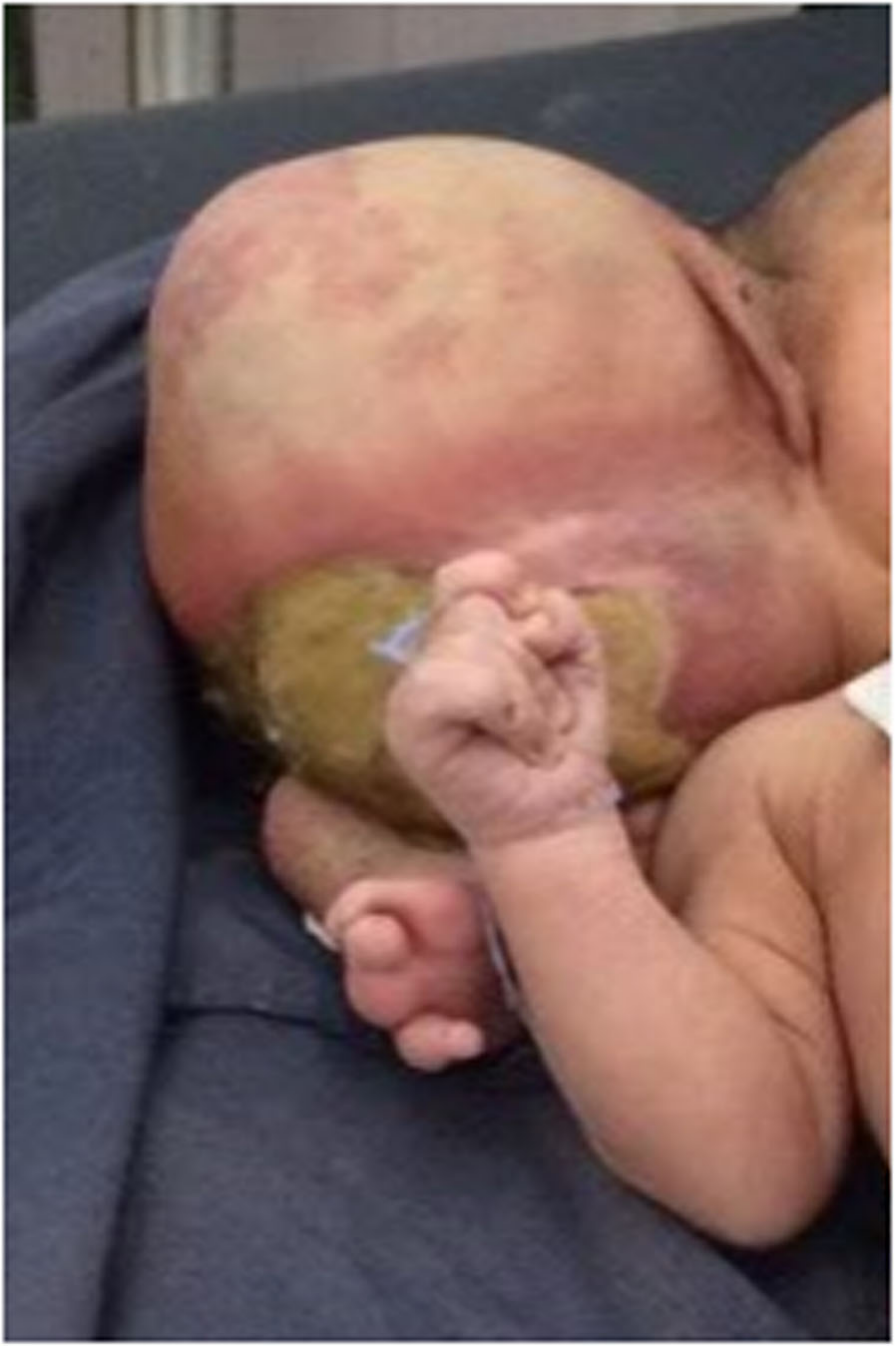
Autosite (normal twins) with parasitic twin after delivery

**Fig. 4:**
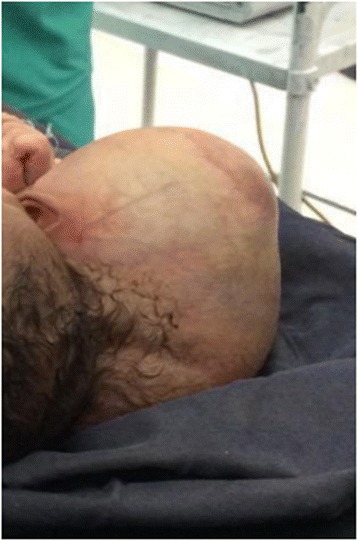
Top view of Craniopagus Parasiticus

**Fig. 5:**
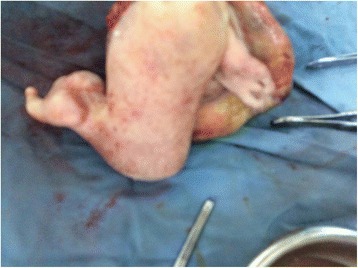
Separated parasitic twin

A cesarean section was done under general anesthesia and a live baby girl weighing 4200 g was delivered. The placenta was single and normal. Her Appearance, Pulse, Grimace, Activity, and Respiration (APGAR) scores were 7 and 9 at 1 and 5 minutes, respectively. She appeared to be grossly normal except her parasitic co-twin was attached at the temporal area of her cranium (see Figs. 1, 2, 3, 4, and 5). Her twin was an incidental finding and during the difficult extraction her left uterine artery was severed and repaired.

The baby girl was further evaluated with a skull X-ray; an ultrasound of the co-twin and the abdomen of the normal twin (autosite) by Doppler ultrasound confirmed that the parasitic conjoined twin had communication with the normal twin only in soft tissue and vessel arising from carotid vessels but no connection with the brain or related structures.

A detailed clinical examination of the normal twin revealed normal findings except for her parasitic twin at her cranial region. All four limbs of the normal twin were moving freely but no movement was detected at the parasitic twin. Auscultation to the heart of the normal twin was normal. The parasitic twin contained disproportionately developed lower limbs that had four toes on each limb. The parasitic twin had no distinctly separable abdomen, chest, or cranium. The parents were counselled and informed by a multidisciplinary team of nurses, anesthesiologists, pediatricians, gynecologists, and surgeons as to the subsequent plan of management.

Surgery was performed to the baby 1 week after her delivery after the necessary investigation and preparation was done. The parasitic co-twin was totally excised in the operation that took approximately 6 hours. Her postoperative period was smooth and uneventful; she comfortably suckled on the breast well. She was transfused with a calculated two units of fresh whole blood. Two weeks after the surgery she was discharged healthy with an arrangement for postnatal follow up.

After separation, a pathologic examination demonstrated that skin covered the body of the parasitic twin. The parasitic twin had two deformed lower limbs, one of which was rudimentary. After dissection of the mass of the body, the intestine was seen but there were no chest organs or abdominal organs. The long bones of the bilateral lower limbs and some pelvic bone were seen in the limbs of the parasitic twin. There was also a rudimentary labium but no vaginal opening.

## Conclusions

Craniopagus parasiticus is an extremely rare condition of parasitic twinning; it is characterized by the conjoining of twins at the head. The primary cause is unclear; genetic scientists are still investigating the development of this condition [7]. In the development of normal monozygotic twins, one egg is fertilized by a single sperm. Then the egg splits into two, frequently during the two-cell stage. If the splitting of the egg occurs during the initial blastocyst phase, two inner cell masses tend to form, consequently the twins share the same placenta and chorion results, but with distinct amnions. It is also possible for the egg to divide into two but have one blastocyst. This results in one blastocyst and one inner cell mass. In such cases, during development the twins have a tendency to share the same chorion, placenta, and amnion. This is the one of the most likely reasons for the occurrence of conjoined twins. It is also probable that such an abnormality has a part in craniopagus parasiticus [8, 9]. On the other hand, it is known that parasitic twins form in the utero and start development in the embryo, but the twins fail to completely split into two. In this condition, the dominant embryo fully develops, while the other embryo’s development is extremely restricted during gestation [2].

One hypothesis for the development of craniopagus parasiticus is that a single zygote leads to the development of two fetuses but separation fails either during the second or fourth week of gestation. This is known as fission theory. Another hypothesis is that craniopagus parasiticus is caused by a lack of blood supply to the second twin brought about by the degeneration of the umbilical cord, thereby halting the development of the fetus [10]. The main difference between a parasitic twin and conjoined twins is that the parasitic twin fails to develop during gestation, while the normal twin develops fully [11].

Only ten cases of craniopagus parasitic twins have been documented in the literature. Recently, Manar Maged, the normal twin of craniopagus parasitic twins, underwent surgery, which is an indication that a normal twin can survive. Manar Maged was able to survive without any signs of paralysis until a few days before her second birthday when she died due to a severe infection in her brain [12].

In our case, a pregnant woman was referred from a rural health center for prolonged second stage labor. On her arrival at our hospital an obstetrician decided to do a caesarean section because she was unable to deliver vaginally. During the procedure, a baby girl weighing 4200 g was delivered but unexpectedly she was a craniopagus parasitic twin; the placenta was single and normal. We evaluated the delivered baby by using a skull X-ray, an ultrasound of the co-twin, and an ultrasound of the abdomen of the normal twin. Doppler ultrasound confirmed that the parasitic conjoined twin had no connection with the brain or related structures of the normal twin, and the only communication was soft tissue and vessels arising from carotid vessels.

After necessary counselling and preparation was completed, a 6-hour successful separation surgery was done 1 week after her birth. Postoperation, she easily suckled breast and had no neurological deficit. Two weeks after separation she was discharged in a good healthy condition with an arrangement for postnatal follow up. A pathological examination of the parasitic twin revealed two deformed lower limbs, one of which was rudimentary. Dissection of the mass of the body showed the intestine but no chest organs or abdominal organs. The long bones of the parasitic twin’s bilateral lower limbs and some pelvic bone were seen. There was a rudimentary labium but no vaginal opening (see Figs. 1, 2, 3, 4, and 5).

In conclusion, the causes of craniopagus parasiticus are still unknown due to a rarity of cases and the limited number of studies on it. There have been only nine or ten cases of craniopagus parasiticus, of which only three survived past birth and were documented in the literature. We hope that genetic scientists and researchers continue to investigate this case because they might find explanations of the birth defect, and provide answers and improve the prognosis and the life chances of twins with craniopagus parasiticus. In our case, the baby girl is in good health and suckling breast milk after a successful separation was performed.

## Abbreviations

APGAR: Appearance pulse grimace activity and respiration
HBV: Hepatitis B virus
HIV: Human immunodeficiency virus
